# Novel insights into exosomal circular RNAs: Redefining intercellular communication in cancer biology

**DOI:** 10.1002/ctm2.636

**Published:** 2021-12-12

**Authors:** Huimin Lin, Jie Yu, Xiang Gu, Shengfang Ge, Xianqun Fan

**Affiliations:** ^1^ Department of Ophthalmology Ninth People's Hospital Shanghai JiaoTong University School of Medicine Shanghai 20025 P. R. China; ^2^ Shanghai Key Laboratory of Orbital Diseases and Ocular Oncology Shanghai 20025 P. R. China

**Keywords:** biomarker, cancer, circular RNAs, exosomal circRNAs, exosomes

## Abstract

Exosomes, a special type of membrane‐bound extracellular vesicle regarded as an ideal carrier for intercellular messages, play an essential role in intercellular communication both locally and systematically. Recent studies have reported that circular RNAs (circRNAs), members of the noncoding RNA family, are abundant and stable in exosomes. As an essential mediator of intercellular communication within cancer cells or between cancer cells and noncancerous cells, exosomal circRNAs participate in multiple aspects of cancer. In this review, we summarize the biogenesis, properties and functions of exosomal circRNAs. In particular, we describe their intercellular transfer in the tumour microenvironment and associate their biological functions with different phenotypes of cancer. Finally, we discuss potential clinical applications in the future.

## Graphical abstract

1. Circular RNAs (circRNAs) can exert their functions via exosome delivery to recipient cells.
2. Exosomal circRNAs are involved in tumour‐to‐tumour crosstalk, tumour‐to‐stroma crosstalk and stroma‐to‐tumour crosstalk in the tumour microenvironment.
3. Exosomal circRNAs are promising diagnostic, prognostic and predictive biomarkers for cancer, and exosomes serve as vehicles to deliver therapeutic circRNAs.

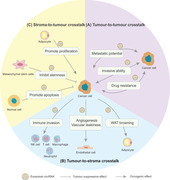

## 1 BACKGROUND

Exosomes are nanoscale (30–150 nm) vesicles of endocytic origin, which are secreted by all types of cells and are enriched in a variety of body fluids such as blood, urine and saliva.^1^ Although mistakenly considered to be cell debris and underestimated for many years, exosomes are now attracting increasing attention as vehicles of intercellular communication.^2^, ^3^ As a special type of extracellular vesicle (EV), exosomes encapsulate and transfer bioactive molecules to target cells, which are involved in a set of physiological and pathological processes, particularly cancer initiation and metastasis.^4^, ^5^, ^6^ The currently studied functional exosomal cargoes encompass a variety of proteins, lipids, DNA, messenger RNAs (mRNAs) and noncoding RNAs, including microRNAs (miRNAs), long noncoding RNAs (lncRNAs) and circular RNAs (circRNAs).^1^, ^7^, ^8^ As an important part of the noncoding RNA family, circRNAs are characterized by a covalently closed loop structure with no 5′ caps and 3′ poly (A), which have high stability, abundance, prevalence and conservation.^9^ Based on differences in the composition and cycling mechanism, circRNAs are classified into three types: exonic circRNAs, intronic circRNAs and exon‐intron circRNAs.^10^ Physiologically, circRNAs act as miRNA sponges, protein sponges or decoys, templates for translation and RNA transcriptional regulators. Pathologically, circRNAs act as both oncogenes and tumour suppressors by regulating cell proliferation, drug resistance, angiogenesis, metabolism, metastasis and antitumour immunity.

In the past few decades, an increasing number of studies have focused on the roles of exosomal miRNAs and lncRNAs in cancer biology and their potential as biomarkers for disease diagnosis or targets for treatment.^11^, ^12^, ^13^ However, benefiting from the advances in high throughput RNA sequencing and analytical methods, circRNAs are proven to be present in exosomes, with high abundance and stability.^14^ Transferred by exosomes, exosomal circRNAs retain their own unique features and participate in the complicated intercellular communication within the tumour microenvironment.^15^ Moreover, the characteristics of exosomal circRNAs may indicate equal or even more important functions in different aspects of cancer biology, and the combination of different exosomal RNA cargoes may have increasing clinical value in the future.

## 2 BIOGENESIS AND FUNCTIONS OF EXOSOMES

The biogenesis of exosomes starts with the invagination of the cytomembrane to form early‐sorting endosomes, which eventually mature into late‐sorting endosomes (LSEs).^16^ Another invagination then occurs. The endosomal limiting membrane of LSEs buds inward and pinches off to form intraluminal vesicles (ILVs), which package a variety of cargoes. LSEs are also called multivesicular bodies (MVBs) because they contain multiple ILVs. Two possible mechanisms have been proposed to explain the formation of MVBs: an endosomal sorting complex required for transport (ESCRT) mechanism and an ESCRT‐independent mechanism. The ESCRT mechanism, which is the more common mechanism, is mediated by a set of cytoplasmic protein complexes called the ESCRT that sort ubiquitinated proteins into the ILVs of MVBs,^17^ while the ESCRT‐independent mechanism is mediated by ceramide^18^ and the tetraspanin CD63.^19^ After the formation of MVBs, MVBs that are destined for the lysosomal pathway fuse with lysosomes, and their contents are degraded and recycled. Alternatively, MVBs also fuse with the plasma membrane and are secreted as exosomes into the extracellular environment. Rab GTPases such as Rab11/35 and Rab27 are involved in MVB trafficking to and docking at the plasma membrane,^20^ and the soluble *N*‐ethylmaleimide‐sensitive factor attachment protein receptor complex is involved in membrane fusion and secretion of exosomes.^21^, ^22^

Exosomes were first proposed as a tool to eliminate unnecessary waste from cells and maintain cellular homeostasis.^23^ However, recent studies have indicated that exosomes are crucially involved in cell–cell communication by delivering the abovementioned functional cargoes.^16^, ^24^ Exosomes transport heterogeneous cargoes to target cells in nearby or distant areas through direct membrane fusion, binding to membrane receptors, and endocytosis,^25^ thus initiating or inactivating multiple signalling pathways. In the development of cancer, exosomes participate in premetastatic niche formation and promote tumour metastasis by functioning as a communication medium between primary tumour sites and distant organs.^26^, ^27^ Exosomes are also involved in tumour microenvironment remodelling, immune evasion, angiogenesis and therapeutic resistance.^5^, ^28^

## 3 BIOGENESIS, PROPERTIES AND FUNCTIONS OF circRNAs

circRNAs are produced from precursor mRNAs through a noncanonical back‐splicing process, which is controlled by both cis‐regulatory elements and trans‐acting factors.^9^ In the back‐splicing process, a downstream splice donor site attacks an upstream splice acceptor splice site to generate a 3′–5′ phosphodiester bond and the circular structure of circRNAs.^29^ circRNAs have several unique properties. First, the covalently closed circular structure makes circRNAs more stable and resistant to ribonucleases, resulting in a longer half‐life period than that of their linear counterparts.^30^ Second, circRNAs are widely expressed in various cell types and organs in humans.^31^ Third, the expression patterns of circRNAs are tissue‐specific, cell type–specific, age‐specific and conserved across mammals.^32^, ^33^, ^34^

circRNAs have four functions: serving as miRNA sponges, binding to proteins (acting as protein sponges or decoys), translating into proteins and promoting linear RNA transcription. The most well‐studied function of circRNAs is the miRNA sponging effect. The competitive endogenous RNA (ceRNA) hypothesis indicates that RNA competes for miRNAs through miRNA response elements and regulates the expression of miRNA‐related genes.^35^ The ceRNA family includes mRNAs, lncRNAs, pseudogenes and now circRNAs. Notably, many circRNAs contain a variety of miRNA binding sites and interact with the corresponding miRNAs, thereby inhibiting miRNA expression and increasing the expression of miRNA‐associated target genes. Researchers first identified a highly expressed circRNA called ciRS‐7 in the human and mouse brains that contained 70 conserved miRNA binding sites.^36^, ^37^ Similarly, circRNA sex‐determining region Y with testis specificity was found to contain 16 binding sites and sponge miR‐138.^37^ Taken together, these studies indicate that the miRNA sponging effect of circRNAs may be a common phenomenon. Moreover, some other functions of circRNAs are also being discovered.^10^ For example, circRNAs function as protein sponges, which harbour many RNA binding protein binding sites and block protein activity. Some circRNAs are also translated into specific proteins because they contain an open reading frame and a binding site of ribosome.^38^ Some circRNAs can promote RNA transcription by interacting with U1 small nuclear ribonucleoprotein and promoting the interaction between RNA polymerase II and the host gene promoter region.^36^ Because of their unique properties and variety of important functions, circRNAs are involved in tumour initiation and progression through diverse pathways.^39^, ^40^ Different types of circRNAs may function as either oncogenes or tumour suppressors in cancer, and even the same circRNAs may play completely opposite roles in different cancer types.^10^ Thus, an understanding of the detailed molecular mechanisms of circRNAs is necessary to elucidate the diverse regulatory roles of circRNAs in cancer.

## 4 THE ROLES OF circRNAs IN EXOSOMES

**FIGURE 1:**
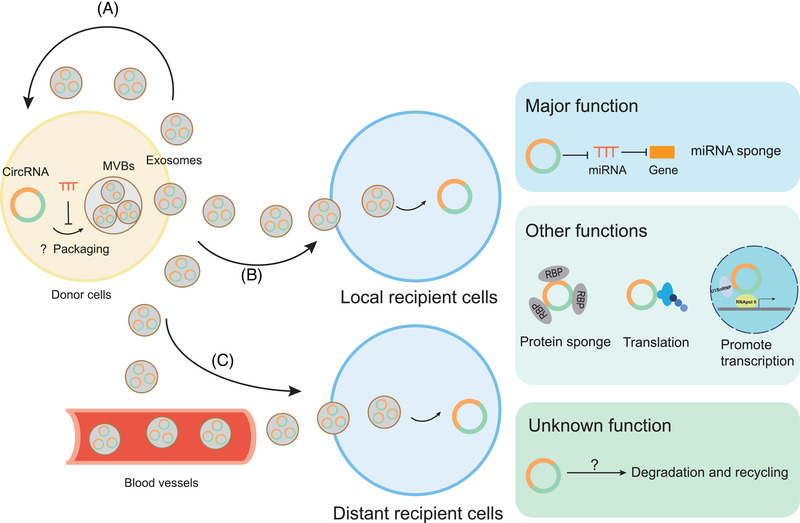
The packaging, delivery and functions of exosomal circular RNAs (circRNAs); circRNAs are selectively packaged and incorporated into exosomes and this process may be actively regulated. The circRNA–microRNA (miRNA) interactions may impact the packaging of circRNAs into exosomes. However, the detailed mechanism remains unknown. Then exosomes derived from donor cells can be delivered to themselves in an autocrine manner (A), to local recipient cells in a paracrine manner (B) or through blood vessels to distant recipient cells in an endocrine manner (C). The major function of the taken‐up exosomal circRNA is to act as a sponge of miRNA and abolish the silencing effect of miRNAs on target genes, thus enhancing the expression of miRNA‐related genes. Other functions of exosomal circRNAs may include acting as a protein sponge, translating into protein and promoting transcription, which is still being illustrated. Exosomal circRNAs may also be degraded and recycled, which has not been fully confirmed

In 2015, Li first reported that circRNAs were enriched and stable in exosomes and they might represent a novel biomarker for cancer diagnosis, which paved the way for subsequent studies on exosomal circRNAs.^14^ To date, the packaging and delivery mechanisms and the functions of circRNAs in exosomes are gradually being revealed (Figure 1). circRNAs are selectively packaged and incorporated into exosomes, and this process may be actively regulated.^41^ Recent reports have observed the differential expression patterns of circRNAs between exosomes and the cytoplasm. A study of three colorectal cancer (CRC) cell lines showed that circRNAs were downregulated in mutant KRAS cell lines, while circRNAs were upregulated in exosomes secreted from the corresponding cells, providing evidence that cytoplasmic circRNAs may be selectively transported into exosomes.^42^ Another recent study observed the selective release of circRNAs into exosomes derived from platelets.^43^ Compared with the linear isoforms, the circular isoforms of FAM13B, DYRK1A, AMD1 and TMEM30 were preferentially released into exosomes, while the circular isoform of ASAP1 was preferentially retained in platelets. The results also implied that the potential sorting determinants might include the size and particular sequence motifs of circRNAs. Moreover, the level of exosomal circRNAs might be associated with changes in relevant miRNA expression, indicating that circRNA–miRNA interactions might alter the packaging of circRNAs into exosomes.^14^ So far, the underlying mechanisms of circRNA sorting into exosomes are still unknown and require further investigation. After their release into the extracellular environment, exosomes derived from donor cells are delivered to these cells in an autocrine manner.^44^ However, the main destinations of exosomes are local or distant recipient cells. circRNAs packaged in exosomes can be delivered to local recipient cells in a paracrine manner or released into blood vessels to travel to distant recipient cells in an endocrine manner.^45^

Many aspects of the functions of circRNAs in exosomes have been identified. On the one hand, exosomes serve as ideal messengers through which functional circRNAs travel long or short distances and modulate the biological behaviour of target cells, mediating cell–cell communication. Because of the protection of exosomes, the circular nature and biological activity of circRNAs are maintained during their transport toward target cells.^14^ After uptake into target cells, exosomal circRNAs exert their functions, the most well‐known of which is the miRNA sponge effect.^46^ However, as circRNAs have been gradually shown to have other functions, such as acting as protein sponges, translating into proteins and promoting transcription, exosomal circRNAs are also predicted to have these functions. Among them, the protein sponge effect is confirmed. For example, circSHKBP1 transported by exosomes to gastric cancer cells not only regulates the miR‐582‐3p/HUR/vascular endothelial growth factor (VEGF) axis but also sponges the HSP90 protein and blocks the interaction with STUB1 of HSP90 to inhibit the ubiquitination of HSP90.^47^ For another example, exosomal circ‐CCAC1 sponges the EZH2 protein to sequester EZH2 in the cytoplasm and blocks EZH2‐mediated promoter H3K27 trimethylation of SH3GL2.^48^ However, direct evidence for the other two functions of exosomal circRNAs is lacking. On the other hand, exosomes may be a way for cells to eliminate excess or unnecessary cytoplasmic circRNAs, which are destined for degradation and recycling.^49^ A study observed the enrichment of circRNAs in exosomes compared with the cell body relative to their linear counterparts from three different cell lines and proposed that exosomes might represent a potential circRNA clearance method in cells.^50^ However, direct evidence for exosome‐mediated circRNA clearance is still lacking and merits further investigation.

## 5 THE DATABASE OF EXOSOMAL circRNAs

Many databases on circRNAs have been established, but databases on exosomal circRNAs are few. Currently, two databases, ExoRBase and BBCancer, have been established. ExoRBase is the first exosome‐based database to provide detailed information on all available long RNA species in human blood exosome samples, containing 58 330 circRNAs, 15 501 lncRNAs and 18 333 mRNAs.^51^ The collected blood exosome samples are derived from individuals with different biological conditions, including both healthy individuals and individuals with different diseases, such as coronary heart disease, hepatocellular carcinoma (HCC), CRC, pancreatic adenocarcinoma and breast cancer. Particularly, the annotation, expression levels and potential original tissues of exosomal circRNAs are provided. BBCancer is a blood‐based database that provides the expression profiles of six types of RNA molecules (circRNAs, lncRNAs, miRNAs, mRNAs, tRFRNAs and piRNAs) in blood samples, including plasma, EVs and circulating tumour cells.^52^ This database contains data on 60 306 circRNAs in EVs collected from healthy volunteers and patients with CRC, liver cancer and pancreatic cancer. The two databases described above provide comprehensive landscapes of blood exosome‐based circRNAs, together with circulating circRNAs or other exosomal RNA molecules, which would be beneficial to developing potential biomarkers for cancer.

## 6 THE INTERCELLULAR COMMUNICATION OF EXOSOMAL circRNAs IN CANCER BIOLOGY

**FIGURE 2:**
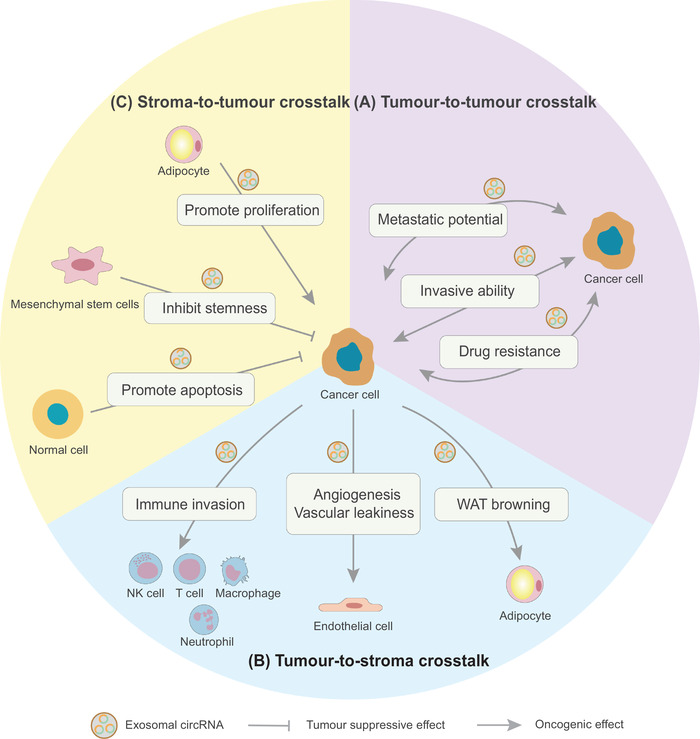
Exosomal circular RNA (circRNA)–mediated intercellular communication in cancer biology; exosomal circRNAs act as oncogenes or tumour suppressors in the development of cancer. (A) In tumour‐to‐tumour crosstalk, exosomal circRNAs are transferred from cancer cells with highly invasive ability, metastatic potential or drug resistance to less malignant cancer cells, contributing to an increase in cancer cell malignancy. (B) In tumour‐to‐stroma crosstalk, exosomal circRNAs are transferred from cancer cells to stroma cells, such as immune cells, endothelial cells and adipocytes, resulting in the construction of a tumour‐promoting microenvironment. (C) In stroma‐to‐tumour crosstalk, exosomal circRNAs are transferred from stroma or normal cells to cancer cells to regulate the behaviour of tumour cells

Different types of circRNAs and circRNAs transported by exosomes function as either oncogenes or tumour suppressors in the development of cancer. Exosomal circRNAs are involved in multiple aspects of tumour progression by participating in the complex intercellular communication network of cancer cells, stromal cells and normal cells in the tumour microenvironment (Figure 2). The intercellular communication of exosomal circRNAs has been divided into three parts. First, exosomal circRNAs are transferred between cancer cells, which is called tumour‐to‐tumour crosstalk. Cancer cells are heterogeneous in both phenotypes and biological functions within the same tumour tissue because of genetic changes and environmental differences.^53^ Cancer cells with highly invasive and metastatic potential or drug resistance transmit exosomal circRNAs to less malignant cancer cells, contributing to an increase in cancer cell malignancy. Typical examples are circPTGR1 and circRNA‐SORE, which will be described in detail later. Briefly, exosomal circPTGR1 is transferred among cancer cells to spread metastatic ability, and exosomal circRNA‐SORE is transferred among cancer cells to spread sorafenib resistance.^54^, ^55^ Second, exosomal circRNAs are transferred from cancer cells to stromal cells, which is known as tumour‐to‐stroma crosstalk. The growth, invasion and progression of tumours is supported by the construction of the tumour microenvironment, which comprises diverse cell types, including both cancer cells and stromal cells, such as endothelial cells, pericytes, immune cells, fibroblasts and adipocytes.^56^ Cancer cells transfer exosomal circRNAs to these different types of stromal cells, resulting in the formation of the tumour microenvironment, which promotes tumour metastasis, angiogenesis, vascular permeability, immune invasion and other hallmarks of cancer. As two typical examples that will be discussed in detail later, exosomal circ‐CCAC1 is transferred into endothelial cells to promote vascular permeability and angiogenesis and exosomal circUHRF1 is transferred into natural killer (NK) cells to elicit NK cell exhaustion.^48^, ^57^ Third, exosomal circRNAs derived from stromal cells and normal cells inversely modulate the behaviour of cancer cells, which is also called stroma‐to‐tumour crosstalk. The ability of exosomal circRNAs to inhibit or promote cancer cell growth depends on the types of donor cells and circRNAs. For example, exosomal circ_0030167 derived from bone marrow mesenchymal stem cells inhibits the malignant progression and stemness of pancreatic cancer cells through the miR‐338‐5p/wif1/wnt8/β‐catenin axis.^58^ In contrast, circ‐DB derived from adipocytes promotes the growth of HCC cells through the miR‐34a/USP7/cyclin A2 signalling pathway

**FIGURE 3:**
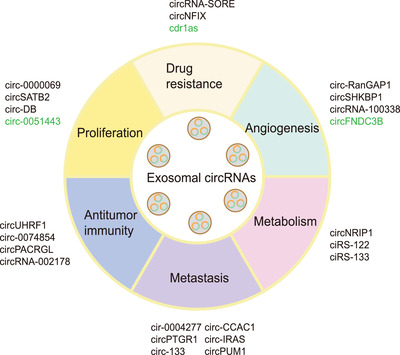
The association between exosomal circular RNAs (circRNAs) and different tumour phenotypes; different types of exosomal circRNAs are associated with different tumour phenotypes, including proliferation, drug resistance, angiogenesis, metabolism, metastasis and antitumour immunity. Tumour‐suppressive exosomal circRNAs are indicated in green and oncogenic exosomal circRNAs are indicated in black

Recent studies have reported the associations between exosomal circRNAs and common tumour phenotypes, including proliferation, drug resistance, angiogenesis, metabolism, metastasis and antitumour immunity (Figure 3). Here, we have summarized recent reports on the roles of exosomal circRNAs in cancer and focused on their delivery between cells as well as biological functions (Table 1).

**TABLE 1:** The roles of exosomal circular RNAs (circRNAs) in cancer

Cancer phenotypes	Exosomal circRNAs	Cancer types	Donor cells	Recipient cells	Functions in cancer	Reference
Proliferation	circ‐0000069	PDAC	PDAC cells	Pancreatic duct epithelial cells	Promote cell proliferation and cell cycle through the miR‐144/STIL pathway	^60^
	circSATB2	NSCLC	NSCLC cells	NSCLC cells and normal bronchial epithelial cells	Promote tumour progression and induce the malignant transformation of normal cells through the miR‐326/FSCN1 pathway	^61^
	circ‐0051443	HCC	Normal cells	HCC cells	Promote cell apoptosis and arrest cell cycle through the miR‐331‐3p/BAK1 pathway	^63^
	circ‐DB	HCC	Adipocytes	HCC cells	Promote tumour growth and decrease DNA damage through the miR‐34a/USP7/cyclin A2 pathway	^64^
Drug resistance	circRNA‐SORE	HCC	Sorafenib‐resistant HCC cells	Sorafenib‐sensitive HCC cells	Spread sorafenib resistance through interaction with YBX1	^55^
	circNFIX	Glioma	Temozolomide (TMZ)‐resistant glioma cells	TMZ‐sensitive glioma cells	Spread TMZ resistance through sponging miR‐132	^65^
	cdr1as	Ovarian cancer	Serum samples	‐	Enhance cisplatin chemosensitivity through the miR‐1270/SCAI pathway	^66^
Angiogenesis	circ‐RanGAP1	GC	Plasma samples	‐	Promote angiogenesis and metastasis through the miR‐877‐3p/vascular endothelial growth factor (VEGF) A pathway	^69^
	circSHKBP1	GC	Serum samples	‐	Promote angiogenesis and metastasis through the miR‐582‐3p/HUR/VEGF pathway	^47^
	circFNDC3B	CRC	CRC cells	CRC cells	Inhibit angiogenesis through the miR‐937‐5p/tissue inhibitors of the metalloproteinases 3 pathway	^71^
	circRNA‐100338	HCC	HCC cells	Human umbilical vein endothelial cells	Promote angiogenesis, vascular permeability and vasculogenic mimicry formation ability	^72^
Metabolism	circNRIP1	GC	GC cells	GC cells	Alter metabolism and autophagy and promote tumour metastasis through the miR‐149‐5p/AKT1/mTOR pathway	^74^
	ciRS‐122	CRC	Oxaliplatin‐resistant CRC cells	Oxaliplatin‐sensitive CRC cells	Promote glycolysis and induce resistance to oxaliplatin through the miR‐122/pyruvate kinase M2 isoform pathway	^75^
	ciRS‐133	GC	GC cells	Preadipocytes and adipocytes	Promote white adipose tissue browning of preadipocytes and regulate metabolic activity of adipocytes through the miR‐133/PRDM16 pathway	^76^
Metastasis	circ‐0004277	HCC	Normal cells	Human hepatic cells	Enhance the epithelial–mesenchymal transition and migration in HCC and normal cells by inhibiting ZO‐1	^78^
	circPTGR1	HCC	Higher metastatic HCC cells	Lower or nonmetastatic HCC cells	Promote migratory and invasive potential of tumour cells through the miR449a/MET pathway.	^54^
	circ‐133	CRC	Hypoxic CRC cells	Normoxic CRC cells	Promote tumour metastasis through the miR‐133a/GEF‐H1/RhoA pathway	^80^
	circ‐CCAC1	CCA	CCA cells	Endothelial monolayer cells	Promote tumour progression, vascular leakage and angiogenesis through sequestering EZH2 in the cytoplasm and upregulating SH3GL2	^48^
	circ‐IRAS	PDAC	PDAC cells	Human microvascular vein endothelial cells	Increase endothelial monolayer permeability and promote tumour invasion and metastasis through the miR‐122/RhoA pathway	^82^
	circPUM1	Ovarian cancer	Ovarian cancer cells	Peritoneal mesothelial cells	Promote the mesothelial‐to‐mesenchymal transition and peritoneal metastasis through the miR‐615‐5p, miR‐6753‐5p/NF‐kB and MMP2 pathway	^83^
Antitumour immunity	circUHRF1	HCC	HCC cells	Natural killer (NK) cells	Suppress NK cell function and cause resistance to anti‐PD‐1 immunotherapy through the miR‐449c‐5p/TIM‐3 pathway	^57^
	circ‐0074854	HCC	HCC cells	Macrophages	Induce M2 macrophage activation	^85^
	circPACRGL	CRC	CRC cells	Neutrophils	Promote the switch of neutrophils from N1 to N2 through the miR‐142‐3p and miR‐506‐3p/transforming growth factor‐β1 pathway	^87^
	circRNA‐002178	Lung adenocarcinoma	Lung adenocarcinoma cells	CD8^+^ T cells	Promote immune evasion through the miR‐34/PDL1 and miR‐28‐5p/PD‐1 pathway	^89^

Abbreviations: CCA, cholangiocarcinoma; CRC, colorectal cancer; GC, gastric cancer; HCC, hepatocellular carcinoma; NSCLC, non‐small‐cell lung cancer; PDAC, pancreatic ductal adenocarcinoma.

### 6.1 Exosomal circRNAs and proliferation

Sustained proliferation is the most fundamental hallmark of cancer cells. The growth of normal cells is tightly regulated, while cancer cells lose the control mechanism and acquire the ability to sustain proliferation through the alteration of cell cycle proteins and constitutive activation of proliferation‐related signal transduction pathways.^59^

**FIGURE 4:**
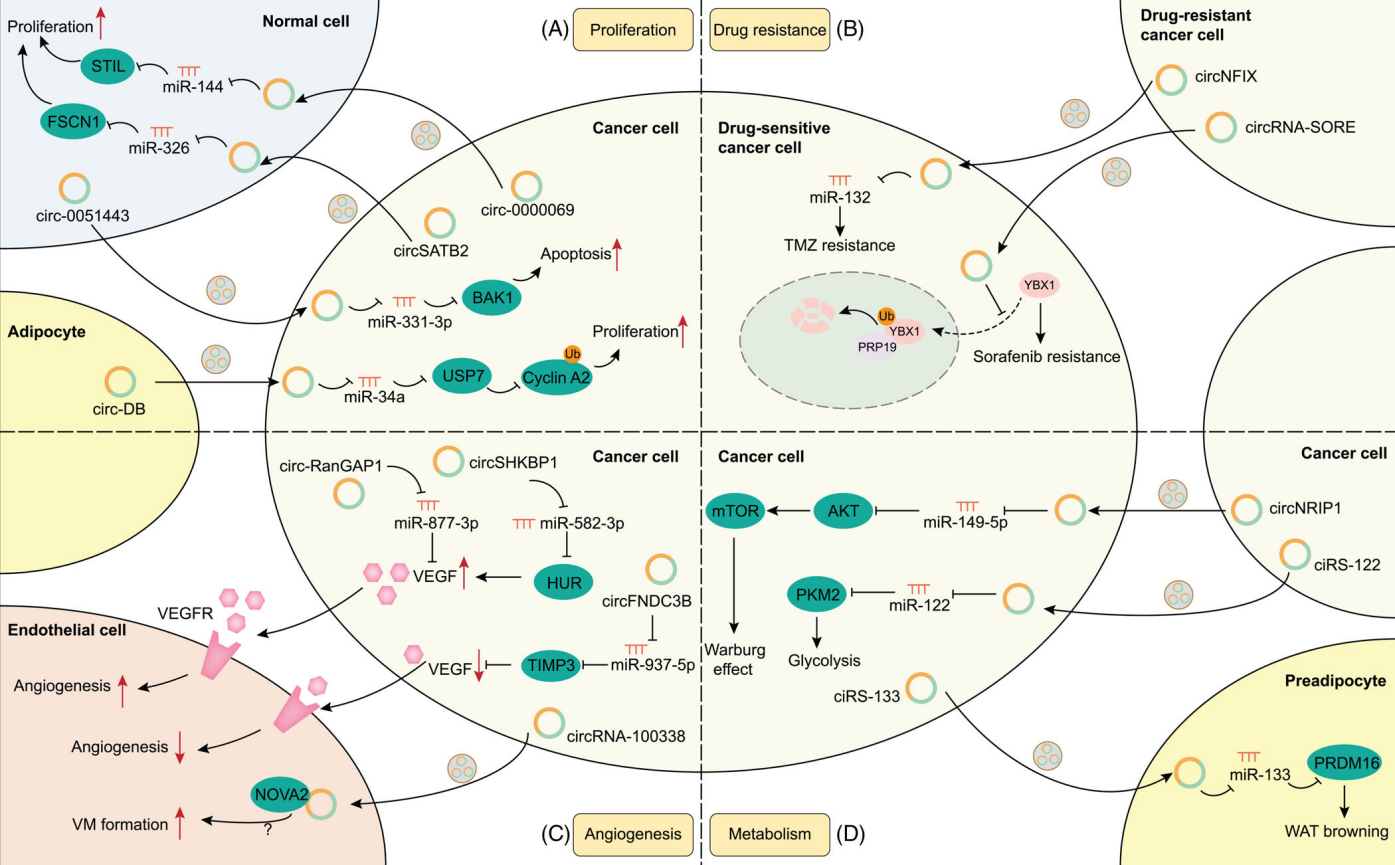
Molecular mechanisms of exosomal circular RNAs (circRNAs) in proliferation, drug resistance, angiogenesis and metabolism; (A) exosomal circRNAs are involved in proliferation. Exosomal circRNAs derived from cancer cells are delivered to normal cells to upregulate STIL and FSCN1 and promote cell proliferation (circ‐0000069, circSATB2, etc.). Exosomal circRNAs derived from normal cells are delivered to cancer cells to upregulate BAK1 and promote apoptosis (e.g., circ‐0051443, etc.) while exosomal circRNAs derived from adipocytes are delivered to cancer cells to activate the USP7/cyclin A2 pathway and promote proliferation (e.g., circ‐DB, etc.). (B) Exosomal circRNAs are involved in drug resistance. Exosomal circRNAs are delivered from drug‐resistant cancer cells to sensitive cells to spread drug resistance through the circNFIX/miR132 pathway and the interaction between circRNA‐SORE and YBX1 to prevent YBX1 nuclear translocation as well as PRP19‐mediated YBX1 ubiquitination and degradation. (C) Exosomal circRNAs are involved in angiogenesis. Exosomal circRNAs not only signal in cancer cells to regulate vascular endothelial growth factor expression and affect angiogenesis (circ‐RanGAP1, circSHKBP1, circFNDC3B, etc.) but are also delivered to endothelial cells and affect vasculogenic mimicry formation through the interaction between circRNA‐100338 and NOVA2 (which still needs further verification). (D) Exosomal circRNAs are involved in metabolism. Exosomal circRNAs are delivered between cancer cells, thus activating the AKT/mTOR pathway to promote the Warburg effect (e.g., circNRIP1, etc.) and elevating pyruvate kinase M2 isoform to promote glycolysis (e.g., ciRS‐122, etc.). Exosomal circRNAs are also delivered from cancer cells to preadipocytes to promote white adipose tissue browning (e.g., ciRS‐133, etc.)

In tumour‐to‐tumour crosstalk, exosomal circRNAs are delivered from cancer cells to neighbouring normal cells or less malignant cells, thus epigenetically regulating the proliferative signalling of recipient cells and contributing to the aberrant growth of recipient cells (Figure 4A). Coculture experiments in vitro have demonstrated that exosomal circ‐0000069 is secreted by pancreatic cancer cells and is internalized by normal pancreatic duct epithelial cells. Exosomal circ‐0000069 promotes cell proliferation and cell cycle and induces the malignant transformation of normal cells by sponging miR‐144 and upregulating STIL, which regulates centriolar replication and propels cell cycle.^60^ Another study has identified that exosomal circSATB2 is delivered to both normal human bronchial epithelial cells and lung cancer cells, where it induces the aberrant proliferation of normal cells and cancer cells by sponging miR‐326 and upregulating FSCN1 expression.^61^

In stroma‐to‐tumour crosstalk, exosomal circRNAs derived from stromal or normal cells also epigenetically regulate the proliferation of cancer cells. In the early stages of tumourigenesis, noncancerous cells release exosomes containing growth‐inhibitory circRNAs to surrounding newly transformed cancerous cells and result in cell apoptosis and growth inhibition, which is a homeostasis mechanism.^62^ In HCC, exosomal circ_0051443 derived from normal liver cells promotes cell apoptosis, arrests the cell cycle and inhibits the malignant phenotype of HCC cells by sponging miR‐331‐3p and upregulating BAK1, a cell death regulator that initiates mitochondria‐mediated cell apoptosis.^63^ However, when this balance between normal cells and cancerous cells is disturbed, the tumour microenvironment is prone to support neoplasms. For example, exosomal circ‐DB derived from adipocytes is delivered to HCC cells and promotes tumour growth via sponging miR‐34a and upregulating USP7, which inhibits the ubiquitination of cell cycle protein cyclin A2 and thus upregulates the level of cyclin A2.^64^

### 6.2 Exosomal circRNAs and drug resistance

Both drug‐resistant and drug‐sensitive cancer cells exist in the early process of tumourigenesis; however, drug‐resistant cells survive as tumours develop. One possible reason is that drug resistance can be transferred through exosomal circRNAs within cancer cells. After the internalization of exosomes from drug‐resistant cancer cells and activation of downstream pathways by circRNAs, cancer cells previously sensitive to drug treatment can acquire drug resistance (Figure 4B). In HCC, circRNA‐SORE is essential for the maintenance of sorafenib resistance by interacting with the oncogenic protein YBX1 in the cytoplasm, preventing the nuclear translocation of YBX1 and subsequently blocking PRP19‐mediated YBX1 ubiquitination and proteasomal degradation in the nucleus.^55^ Moreover, circRNA‐SORE in exosomes is transferred from sorafenib‐resistant cancer cells to sensitive cancer cells, thus facilitating the spread of sorafenib resistance. Another study of glioma has showed that circNFIX promotes temozolomide (TMZ) resistance by sponging miR‐132. Moreover, circNFIX is transmitted by exosomes and confers TMZ resistance from TMZ‐resistant cancer cells to TMZ‐sensitive cancer cells.^65^ However, the downstream target genes of miR‐132 are not mentioned in this work and still need exploration.

Notably, circRNAs also inhibit drug resistance, and dysregulated circRNA expression is reflected in blood exosomes. For example, cdr1as enhances cisplatin chemosensitivity in ovarian cancer cells by sponging miR‐1270 and elevating the expression of the downstream target gene SCAI.^66^ And the cdr1as level is downregulated in tissues and plasma exosomes from cisplatin‐resistant patients. However, there are many unsolved questions remaining to be answered. How can cancer cells downregulate cd1as expression and acquire cisplatin resistance? Can exosomes serve as a way to eliminate tumour‐suppressive cd1as and survive drug‐induced apoptosis? Or can exosomes facilitate the delivery of miR‐1270 to achieve the same SCAI silence effect and spread drug resistance?

### 6.3 Exosomal circRNAs and angiogenesis

Angiogenesis, an important malignant trait of cancer, not only helps cells acquire more nutrients and oxygen but also contributes to the output of metabolic wastes and carbon dioxide.^67^ The angiogenesis switch is governed by activators such as VEGF and inhibitors such as tissue inhibitors of metalloproteinases (TIMP).^68^ Exosomal circRNAs regulate angiogenesis by integrating into angiogenic signalling pathways both in cancer cells and endothelial cells (Figure 4C).

Dysregulated tumour‐derived exosomal circRNAs have been shown to signal in cancer cells and affect the expression of these angiogenic regulator genes in cancer cells. For example, Lu et al. found that exosomal circ‐RanGAP1 promotes tumour angiogenesis by sponging miR‐877‐3p and upregulating VEGFA expression in gastric cancer cells.^69^ As shown in another study by Xie et al., exosomal circSHKBP1 induces angiogenesis by sponging miR‐582‐3p and increasing HUR expression, which increases the mRNA stability and translation of VEGF in gastric cancer cells.^47^ The angiogenic effect can be blocked by bevacizumab, an antiangiogenic drug targeting VEGF. As we know, TIMP3 is a potent angiogenesis inhibitor which blocks the binding of VEGF to VEGF receptors.^70^ CircFNDC3B serves as a sponge for miR‐937‐5p and induces the expression of TIMP3 in CRC cells and circFNDC3B‐enriched tumour‐derived exosomes inhibit angiogenesis in neighbouring CRC cancer cells.^71^

Uptake of tumour‐derived exosomal circRNAs by endothelial cells also directly affects the behaviour of endothelial cells, which is tumour‐to‐stroma crosstalk. Huang et al. reported that exosomal circRNA‐100338 is transferred to human umbilical vein endothelial cells, where it promotes angiogenesis, permeability and vasculogenic mimicry formation.^72^ The proangiogenic effect might be mediated by the interaction with NOVA2, a protein regulating vascular development and lumen formation. However, this hypothesis still needs further verification.

### 6.4 Exosomal circRNAs and metabolism

Sustained tumour proliferation requires adjustments of energy metabolism to fuel cell growth.^73^ Transfer of exosomal circRNAs among cancer cells may cause dysregulated expression of specific metabolic genes and metabolic pathways, thus altering tumour metabolism (Figure 4D). Among them, the AKT1/mTOR axis is a classic metabolic pathway required to sustain tumour metabolic homeostasis. Zhang et al. revealed increased circNRIP1 expression in gastric cells, and it is transported by exosomes between gastric cancer cells, mediating tumour‐to‐tumour crosstalk in the tumour microenvironment.^74^ Exosomal circNRIP1 facilitates energy production (the Warburg effect) and inhibits catabolic activities (autophagy) through sponging of miR‐149‐5p and activation of the AKT1/mTOR signalling pathway. For another example, tumour cells usually rely on aerobic glycolysis to produce energy, which is catalyzed by the pyruvate kinase M2 isoform (PKM2). Exosomal ciRS‐122, which is transmitted among cancer cells, promotes glycolysis and induces drug resistance by sponging miR‐122 and upregulating PKM2.^75^

In tumour‐to‐stroma crosstalk, exosomal ciRS‐133, which has high expression levels in gastric cancer cells, is delivered into preadipocytes and adipocytes.^76^ Exosomal ciRS‐133 then promotes white adipose tissue browning of preadipocytes and regulates the metabolic activity of adipocytes by sponging miR‐133 and upregulating PRDM16 expression. Knockdown of ciRS‐133 leads to reduced production of heat and oxygen consumption, indicating that exosomal ciRS‐133 may play a crucial role in cancer‐associated cachexia, a metabolic syndrome characterized by weight loss and systemic inflammation. This study echoes the abovementioned study, that is, exosomal circ‐DB is delivered from adipocytes to promote cancer cell growth, constituting a complete bidirectional crosstalk between cancer cells and adipocytes.^64^

### 6.5 Exosomal circRNAs and metastasis

**FIGURE 5:**
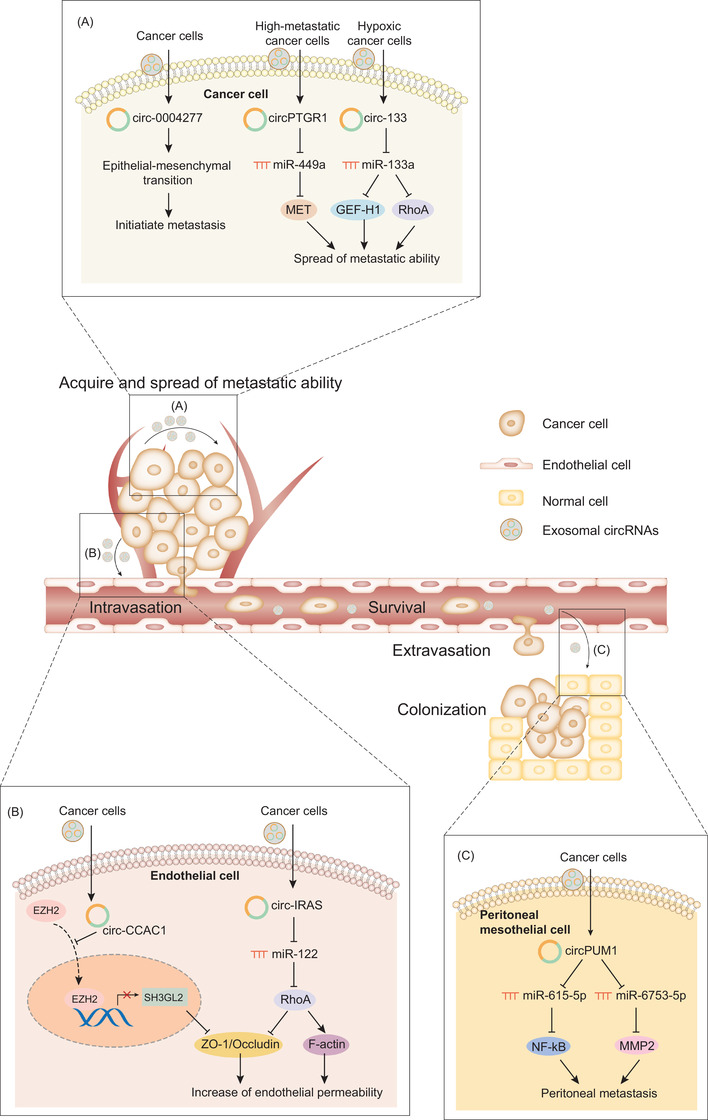
Molecular mechanisms of exosomal circular RNAs (circRNAs) in metastasis; exosomal circRNAs are involved in every step of metastasis, including acquisition and spread of metastatic ability, intravasation into blood vessels, survival and transmission through circulation, extravasation and colonization in distant organs. (A) Cancer cells with high malignancy transmit exosomal circRNAs to neighbouring low malignant cells to promote the epithelial–mesenchymal transition and initiate metastasis (e.g., circ‐0004277, etc.) or spread metastatic ability (circPTGR1, circ‐133, etc.). (B) Cancer cells transmit exosomal circRNAs to endothelial cells to destroy endothelial cell junctions such as ZO‐1 and increase vascular permeability (circ‐CCAC1, circ‐IRAS, etc.). Notably, circ‐CCAC1 prevents EZH2 nuclear translocation and blocks EZH2‐mediated SH3GL2 inhibition, thus upregulating SH3GL2 and decreasing ZO1 and occludin. (C) Cancer cells transmit exosomal circRNAs to distant peritoneal mesothelial cells to promote peritoneal metastasis (e.g., circPUM1, etc.)

Metastasis is a complex multistep process, including acquisition and spread of metastatic potential among cancer cells, local invasion, intravasation of cancer cells into blood vessels, transit through the circulation, extravasation and colonization in distant organs.^77^ Exosomal circRNAs affect every step of the cascade through tumour‐to‐tumour crosstalk and tumour‐to‐stroma crosstalk (Figure 5).

Cancer cells initially acquire the ability to invade and disseminate through epithelial–mesenchymal transition (EMT), in which epithelial cells convert into mesenchymal cells. The EMT process is fuelled by exosomal circRNAs. For example, circ‐0004277 not only enhances the EMT and migration in HCC cells by inhibiting ZO‐1, but is also delivered to neighbouring normal cells through exosome communication and induces their EMT behaviour to promote the malignant transformation of normal cells.^78^

Subsequently, cancer cells spread metastatic potential among tumour subpopulations through tumour‐to‐tumour crosstalk. In this process, exosomal circRNAs are transferred from highly metastatic cancer cells to less metastatic cancer cells, thus epigenetically modulating downstream pathways in recipient cells and facilitating the transfer of metastatic potential between different cancer cell subpopulations. According to Wang et al., HCC cells with a high metastatic ability (LM3 cells) confer this activity to cells with a low metastatic ability (97 L cells) or nonmetastatic cells (HepG2 cells) by delivering exosomes containing circPTGR1, thus promoting the migratory and invasive abilities of cancer cells by competing with miR‐449a and upregulating MET expression.^54^ Moreover, the metastatic potential of cancer cells is relevant to microenvironmental stimuli such as hypoxic conditions. Cancer cells have a heterogeneous oxygen supply even within the same tumour tissues because of the different distances from the blood vessels. Cancer cells under hypoxic conditions are conferred a highly metastatic potential, which can be transferred to relatively normoxic cancer cells through exosomal circRNAs.^79^ In CRC, exosomes secreted from hypoxic cancer cells have recently been shown to contain circ‐133 at high levels and are delivered to normoxic cancer cells. The intercellular transport of exosomal circ‐133 facilitates metastasis by sponging miR‐133a and targeting the GEF‐H1/RhoA axis.^80^

The increase in vascular permeability represents a crucial step before tumour cells intravasate into blood vessels, extravasate from blood vessels and metastasize to distant organs.^81^ Exosomal circRNAs are transferred from cancer cells to endothelial cells, thus weakening endothelial cell junctions and increasing vascular permeability. For example, tumour‐derived exosomal circ‐CCAC1 is transferred to endothelial monolayer cells, thus destroying the integrity of the endothelial barrier.^48^ Mechanistically, exosomal circ‐CCAC1 interacts with EZH2 in the cytoplasm to prevent EZH2 nuclear translocation. Hence, circ‐CCAC1 blocks EZH2‐mediated SH3GL2 inhibition, thus upregulating SH3GL2, which is a negative regulator of two intercellular junction proteins, ZO‐1 and occludin. Another study showed that circ‐IRAS from pancreatic cancer cells is transmitted to human microvascular vein endothelial cells in an exosome‐dependent manner.^82^ When taken up by endothelial cells, circ‐IRAS increases endothelial monolayer permeability and promotes tumour invasion and migration through sponging miR‐122 and upregulating the expression level and activity of RhoA, which further increases F‐actin and decreases ZO‐1.

The last step of metastasis is colonization in distant organs. Exosomal circRNAs are involved in this process through exosome delivery to distant tissues or organs. Exosomal circPUM1 is delivered from ovarian cancer cells to peritoneal mesothelial cells and induces the mesothelial‐to‐mesenchymal transition, thus promoting the peritoneal metastasis of ovarian cancer.^83^ Mechanistically, circPUM1 potentially exerts its function by sponging miR‐615‐5p and miR‐6753‐5p and upregulating NF‐kB and MMP2 and abolishing the inhibitory effects of miRNAs on their targets.

### 6.6 Exosomal circRNAs and antitumour immunity

**FIGURE 6:**
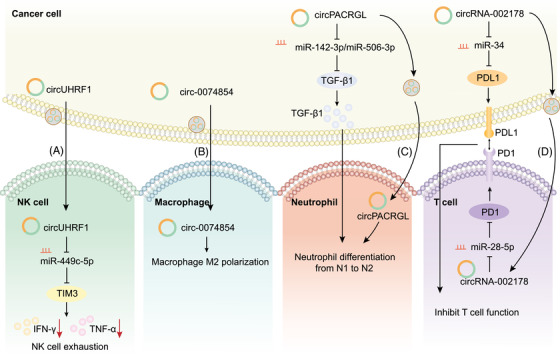
Molecular mechanisms of exosomal circular RNAs (circRNAs) in antitumour immunity; exosomal circRNAs reprogram the biological behaviours of various immune cells. (A) Exosomal circUHRF1 derived from cancer cells is delivered to natural killer (NK) cells to elicit NK cell exhaustion through the miR‐449c‐5p/TIM3 pathway. (B) Exosomal circ‐0074854 derived from cancer cells is delivered to macrophages to induce protumourigenic M2 polarization. (C) CircPACRGL not only increases transforming growth factor‐β1 (TGF‐β1) secretion through the miR‐142‐3p/miR‐506‐3p/TGF‐β1 pathway in cancer cells, but is also delivered to neutrophils through exosomes, collaboratively promoting the differentiation of neutrophils to the protumourigenic N2 phenotype. (D) CircRNA‐002178 not only promotes PDL1 expression in cancer cells by sponging miR‐34, but is also delivered to T cells through exosomes and promotes PD‐1 expression in T cells by sponging miR‐28‐5p. The elevated expression of PD1 and PDL1 inhibits T‐cell function and facilitates tumour immune evasion

Tumour cells manage to evade immune destruction by avoiding detection by the immune system or decreasing the extent of immunological killing. Evidence suggests that exosomes participate in immune evasion by delivering immunosuppressive signals, such as specific circRNAs, to immune cells. In tumour‐to‐stroma crosstalk, tumour‐derived exosomal circRNAs continuously reprogram the biological behaviours of immune cells, such as NK cells, macrophages, neutrophils and T cells, thus creating an immunosuppressive microenvironment favourable for tumour growth (Figure 6).

Exosomal circRNAs disable the normal functions of innate immune cells, typically NK cells, macrophages and neutrophils. For example, exosomes containing circUHRF1 are secreted from HCC cells and delivered into NK cells, which further elicits NK cell exhaustion, inhibits the production of IFN‐γ and TNF‐α and induces resistance to anti‐PD1 therapy by degrading miR‐449c‐5p and upregulating TIM‐3.^57^ Increased levels of plasma exosomal circUHRF1 are also an indicator of decreased NK cell proportions in peripheral blood. Moreover, exosomal circRNAs alter the polarization of macrophages and neutrophils in the microenvironment, facilitating a switch from an anti‐inflammatory phenotype to a protumourigenic phenotype. Macrophages are polarized into M1 or M2 macrophages, and M2 macrophages are related to tumour progression.^84^ Exosomal circ‐0074854 is delivered from HCC cells to macrophages, thereby inducing M2 macrophage activation.^85^ In contrast, exosomes with lower levels of circ_0074854 inhibit M2 macrophage activation and thus suppress tumour migration and invasion. Similarly, N1/N2 neutrophils have various functions, and N2 neutrophils are related to tumour progression.^86^ Exosomal circPACRGL promotes the switch of neutrophils from the anti‐inflammatory N1 phenotype to the protumourigenic N2 phenotype by sponging miR‐142‐3p and miR‐506‐3p and upregulating transforming growth factor‐β1, an immunosuppressive cytokine.^87^

Immune checkpoint molecules such as PDL1/PD1 are critical regulators of immune responses in physiological conditions as well as immune evasion in malignant cancer.^88^ Exosomal circRNAs regulate the behaviours of T cells through PDL1/PD1‐mediated immune evasion. In lung adenocarcinoma, circRNA‐002178 not only promotes PDL1 expression in cancer cells by sponging miR‐34 but is also delivered to CD8^+^ T cells through exosomes and promotes PD1 expression in CD8^+^ T cells via sponging miR‐28‐5p.^89^ Elevated level of PDL1 and PD1 inhibits the function of CD8^+^ T cells and helps cancer cells escape antitumour immune responses. This study has demonstrated that immunosuppressive circRNAs can target PDL1/PD1 in cancer cells and T cells, and exosomes are key bridges for delivering circRNAs from cancer cells to T cells.

## 7 CLINICAL APPLICATIONS OF EXOSOMAL circRNAs

### 7.1 Use of exosomal circRNAs as biomarkers

Exosomes exist in all biological fluids and contain abundant biological molecules, suggesting that they represent a reliable source of cancer‐related molecules (typically miRNAs and now circRNAs) and an ideal sample in liquid biopsies of cancer.^90^ The clinical applications of exosomal miRNAs as tumour biomarkers have attracted wide attention due to their easy and noninvasive sampling methods and high specificity and sensitivity.^91^, ^92^ In addition to miRNAs, emerging evidence has revealed the abundance and stability of circRNAs in tumour‐derived exosomes and focused on the roles of exosomal circRNAs in the diagnosis, prognosis and treatment surveillance of different cancer types. Compared with miRNAs, circRNAs are more stable and resistant to RNase and thus have a longer half‐life. circRNAs are also conserved and have cell‐specific and tissue‐specific expression patterns. Due to these features, exosomal circRNAs may provide a window into altered cellular and extracellular microenvironmental conditions in the cancerous state and appear to represent a novel biomarker for the diagnosis, prognosis and prediction of cancer.^14^, ^93^ First, exosomal circRNAs serve as diagnostic biomarkers that have high sensitivity and specificity. For example, serum exosomal circSATB2 levels are increased in patients with lung cancer compared to healthy volunteers, with an area under the curve (AUC) value of 0.660.^61^ Serum exosomal circRNA‐002178 levels are increased in patients with lung adenocarcinoma compared to healthy volunteers and represent a potential diagnostic biomarker, with an AUC of 0.9956.^89^ Second, exosomal circRNAs serve as prognostic biomarkers. In individuals with pancreatic cancer, plasma exosomal circ‐PDE8A levels are relevant to duodenal and vascular invasion, the tumour node metastasis (TNM) stage and short survival, indicating that it is a promising prognostic biomarker.^94^ In individuals with gastric cancer, plasma exosomal circ‐RanGAP1 levels are relevant to lymph node metastasis and poor clinical outcome, and a prognostic model integrating circ‐RanGAP1 and TNM stage showed an effective prognostic value (AUC, 0.830) compared with individual models.^69^ In individuals with chronic lymphocytic leukaemia, Wu et al. first detected the high abundance of a mitochondrial genome‐derived circRNA called mc‐COX2 in plasma exosomes, and the upregulated exosomal mc‐COX2 levels are relevant to the progression and prognosis of patients.^95^ Third, exosomal circRNAs also serve as biomarkers to predict responses to certain treatments. In individuals with small‐cell lung cancer, Li et al. found that serum exosomal FL1 exonic circRNAs changed dynamically in parallel with the chemotherapy response, indicating its potential as a biomarker to predict therapeutic responses.^96^

The majority of biomarker studies on exosomal circRNAs analyze blood or tissue exosomes. However, exosomal circRNAs in other body fluids also have equal clinical value. In individuals with cholangiocarcinoma, circ‐CCAC1 levels in bile and serum exosomes are not only a potential diagnostic biomarker (AUC values of 0.857 and 0.759, respectively) but also an independent prognostic and recurrent indicator with a possible correlation with adverse clinicopathological characteristics.^48^ In individuals with bladder cancer, the expression level of circPRMT5 is elevated in urine and serum exosomes compared to healthy volunteers, and is an indicator of lymph node metastasis.^97^

### 7.2 Therapeutic circRNA delivery through exosomes

In addition to clinical uses as biomarkers for cancer in liquid biopsy, exosomes can also be modified as a platform to deliver therapeutic circRNAs.^49^ The greatest advantages of exosome‐mediated therapy are the ability of exosomes to protect their nucleic acid cargoes from degradation and deliver the functional cargoes to target cells. Engineered EVs with circSCMH1 overexpression were constructed and combined with rabies virus glycoprotein (RVG) at the surface to realize brain‐specific targeting in subjects with ischemic stroke.^98^ EV‐mediated delivery of functional circSCMH1 improved poststroke recovery in both mice and monkeys, along with an improvement in neuroplasticity and the suppression of glial reactivity and peripheral immune cell infiltration. Small EVs overexpressing sleep‐related circRNA3503 were isolated from synovial mesenchymal stem cells and loaded with the injectable thermosensitive hydrogel PLEL to realize sustained release in individuals with osteoarthritis. The constructed PLEL@circRNA3503‐OE‐sEV delivery system protected cartilage and prevented osteoarthritis progression. However, to the best of our knowledge, the applications of exosome‐mediated tumour‐suppressive circRNA delivery in cancer therapy are lacking and require further investigation.

### 7.3 Perspective on future clinical translation

Some challenges still exist in translating exosomal circRNAs into the clinic. In this part, we list major challenges in future clinical translation and put forward corresponding solutions. In terms of their use as biomarkers, efforts need to be made in realizing technique standardization and improving assay sensitivity and specificity. First, a major hindrance is the lack of standardization in exosome isolation methods (ultracentrifugation, size‐based techniques, immunoaffinity, precipitation and microfluidic) and circRNA profiling methods (RNA‐seq, microarray and quantitative reverse transcription polymerase chain reaction (qRT‐PCR). Consequently, standardized procedures are required in exosomal circRNA research, including blood collection and preservation, exosome isolation and purification and circRNA profiling. With the establishment of a standardized process, the analytical performance and repeatability of specific biomarkers can be evaluated within and between laboratories.^99^ Second, as exosomes are heterogeneous in body fluids and originate from a variety of tissue types, tumour signals are difficult to distinguish from the “noise” of normal cells in RNA transcriptome analyses. Currently, the utilization of immunoprecipitation and nanoflow cytometry to capture specific exosome surface proteins selectively separates exosomes from specific tissues. These techniques enable the enrichment of specific exosomal subpopulations and amplification of disease signals, representing a promising approach to improve the performance of exosome‐based RNA analysis.^100^ Third, further studies should focus on integrating diverse exosomal cargoes (circRNAs, miRNAs, lncRNAs, mRNAs, proteins and metabolites). Currently, a combination of multiple exosomal circRNAs is used to enhance the diagnostic and prognostic accuracy.^101^ However, a combination of exosomal circRNAs with other exosomal cargos is rarely investigated. In fact, the peculiar vesicular structure of exosomes renders them suitable for multianalyte testing. A combinatorial biomarker panel incorporating exosomal circRNAs with other exosomal cargoes that reflect different aspects of exosomes will provide precise and multidimensional information for the diagnosis, prognosis and prediction of cancer.

Challenges also exist in terms of the potential use of exosomes as a delivery system. Major technique limitations are the difficulties in acquiring a purified and well‐characterized exosome population.^49^ In addition, because exosomes are uptaken by different cell types, exosomal circRNAs may also signal in untargeted cells and cause side effects. Consequently, a modified exosomal membrane protein, such as lysosome‐associated membrane glycoprotein 2b (Lamp2b), through linking with targeting peptides or ligands should be developed to target specific tissues or cell types.^102^ In a recent study mentioned above, RVG, which realizes brain‐specific targeting, was engineered at the surface of exosomes through the fusion protein Lamp2b‐RVG.^98^ However, novel cell‐type‐targeting molecules integrating into exosomes are lacking and still require further research.

## 8 CONCLUSIONS

In summary, we discuss the oncogenic and tumour‐suppressive effects of exosomal circRNAs and summarize recent reports on their functions in cancer according to different phenotypes. Moreover, we focus on the complex and fine regulation of intercellular communication between cancer cells and noncancerous cells in the tumour microenvironment mediated by circRNAs packaged in exosomes. We are optimistic that exosomal circRNAs may have high clinical value in the future because the detection of exosomal circRNAs by liquid biopsy may provide novel diagnostic, prognostic and predictive biomarkers for cancer, and the use of exosomes as vehicles to deliver therapeutic circRNAs is promising in future cancer therapy.

## CONFLICT OF INTERESTS

The authors declare that they have no conflict of interests.
